# Cold-sensitive acral hand lesions

**DOI:** 10.1016/j.jdcr.2026.01.009

**Published:** 2026-01-19

**Authors:** Avery H. Seward, Jocelyn H. LaRocque

**Affiliations:** a Eastern Virginia Medical School, Norfolk, Virginia; b Dermatology Care of Charlotte, Charlotte, North Carolina

**Keywords:** chilblains, chilblain lupus, lupus erythematosus

## Case description

An otherwise healthy woman in her 60s presented with a 2-year history of a persistent, itchy, and tender rash on the dorsal hands. The rash fluctuated in severity with the seasons, worsening during the colder months. The patient denied joint pain and fatigue, or lesions elsewhere.

**Fig 1:**
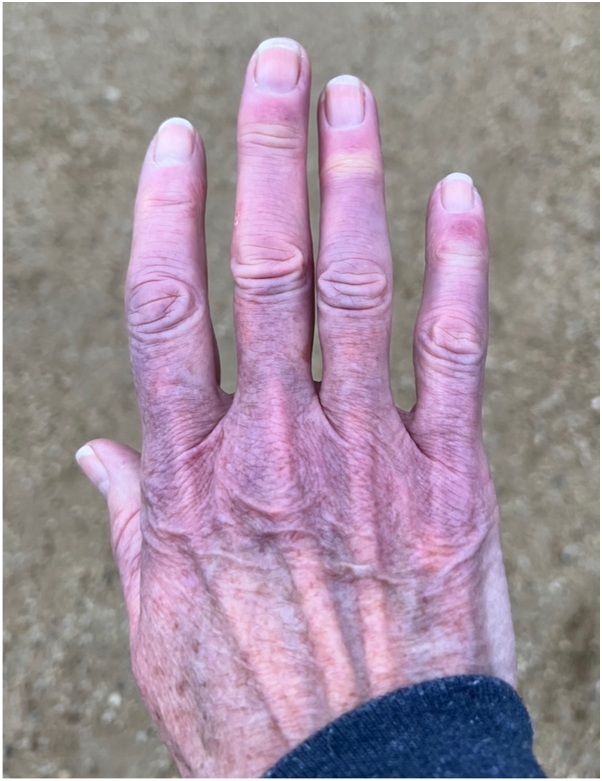
Violaceous plaques on the dorsal hands with partial sparing of the proximal and distal interphalangeal joints.

**Fig 2:**
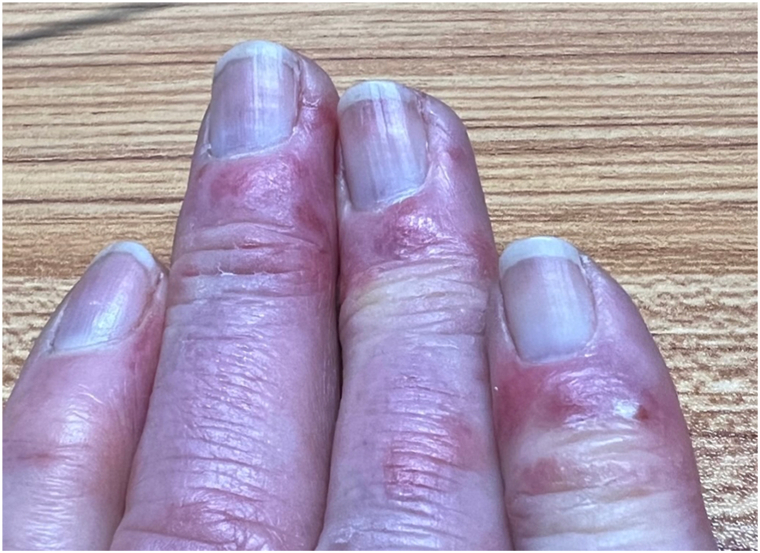
A few punctate, crusted erosions on the dorsal distal phalanges.

**Fig 3:**
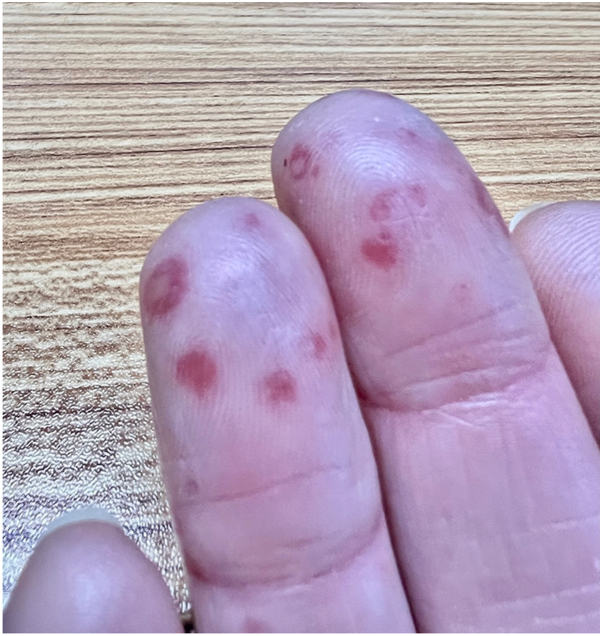
Several purpuric macules on the volar distal phalanges.

Examination revealed partially blanchable violaceous plaques on the dorsal hands with partial sparing of the proximal and distal interphalangeal joints (Fig 1). A few punctate, crusted erosions and purpuric macules were present on the dorsal and volar distal phalanges (Figs 2 and 3).

**Question 1: What is the most likely diagnosis?**

**A.** Acrocyanosis
**B.** Chilblains
**C.** Chilblain lupus
**D.** Raynaud’s phenomenon
**E.** Vasculitis

**Answer:**

**C.** Chilblain lupus

## Discussion

Punch biopsy from the dorsal hand demonstrated a superficial and deep perivascular lymphocytic infiltrate with basal layer squamatization, a smudgy and thickened basement membrane zone on Periodic acid-Schiff, and increased dermal interstitial mucin. Direct immunofluorescence showed granular C5b-9 (membrane attack complex) deposition with weaker IgG deposition and a few cytoid bodies along the epidermal basement membrane. *In vivo* IgG antinuclear antibodies highlighted keratinocyte nuclei.

Antinuclear antibody, anti-double-stranded DNA, anti-Sjogren's Syndrome-related antigen A, anti-Sjogren's Syndrome related antigen B, antiphospholipid antibodies, cryoglobulin, anticentromere B, anti-topoisomerase I antibody, creatine kinase, aldolase, and serum protein electrophoresis were negative. Given the cold sensitivity and acral distribution of the lesions, combined with histology and immunofluorescence supportive of lupus, a diagnosis of chilblain lupus was made.

Chilblain lupus erythematosus (CHLE) is a rare, cold-induced form of cutaneous lupus erythematosus. Chilblains may be primary (idiopathic) or secondary to several conditions including immune-mediated inflammatory disorders, infections (including COVID-19), malignancy, and medications, with systemic lupus erythematosus, the most common secondary association.^1^ Persistence of lesions beyond the colder months may help distinguish CHLE from idiopathic chilblains.^1^, ^2^, ^3^ Because CHLE overlaps clinically with idiopathic chilblains and other cold-induced dermatoses, the Mayo Clinic proposed that a diagnosis of CHLE can be made if 2 major and at least 1 minor criteria are present.^1^

Major criteria:

1. Acral skin lesions associated with cold temperatures.
2. Evidence of lupus erythematosus on histopathology or direct immunofluorescence.

Minor criteria:

1. Coexistence of systemic lupus erythematosus or other skin lesions of discoid lupus erythematosus.
2. Response to antilupus erythematosus therapy.
3. Negative cryoglobulin and cold-agglutinin studies.

CHLE may be sporadic or familial, and both share identical clinical and histopathologic features. Sporadic disease most commonly affects middle-aged women, whereas autosomal dominant familial disease, caused by TREX1 mutations, begins in childhood.^1^ Treatment for CHLE includes cold avoidance, photoprotection, smoking cessation, and topical corticosteroids or calcineurin inhibitors. Nifedipine, pentoxifylline, tadalafil, and antimalarials provide variable success. Systemic corticosteroids or immunosuppressants, and new treatments affecting type-1 interferon, such as anifrolumab and deucravacitinib, may benefit refractory cases.^1^^,^^4^^,^^5^

The patient had previously failed topical corticosteroids and tacrolimus ointment and declined oral nifedipine. Hydroxychloroquine 200 mg twice daily led to significant improvement. The patient continues to use sunscreen and wear gloves as needed.

## Conflicts of interest

None disclosed.
